# Association between nitrogen oxides and COVID-19 risk: A Mendelian randomization study

**DOI:** 10.1097/MD.0000000000046258

**Published:** 2025-11-28

**Authors:** Jing Cao, Haibo Xu, Zixiao Chen, Kun He, Jiaona Wei

**Affiliations:** a The First Department of Pulmonary and Critical Care Medicine, The Second Hospital of Hebei Medical University, Hebei Key Laboratory of Respiratory Critical Care Medicine, Hebei Institute of Respiratory Diseases, Shijiazhuang, Hebei, China.

**Keywords:** COVID-19, Mendelian randomization, nitrogen oxides

## Abstract

Several cross-sectional investigations have indicated an association between nitrogen oxides (NOx) and the spread and fatality of coronavirus disease 2019 (COVID-19); however, none have employed Mendelian randomization (MR). To enhance knowledge of causality, this study examined the association of NOx with COVID-19 risk. Integrated data were obtained from the UK Biobank and integrative epidemiology unit open genome-wide association study for 2-sample MR analysis. Inverse-variance weighting and MR using a robust adjusted profile score were employed for MR analysis, incorporating both multiple random effect and fixed-effect substitution to predict the link between NOx and COVID-19. Furthermore, visual scatter plots, funnel plots, and the leave-one-out method were employed to validate the positive outcomes. MR results revealed that in the inverse-variance weighting method, NOx air pollution exhibited a significant association with COVID-19 (hospitalized vs population; odds ratio = 3.7609; 95% confidence interval = 1.2726–11.1144, *P *= .0166), while nitrogen dioxide (NO_2_) air pollution demonstrated a similar association with COVID-19 (hospitalized vs population; odds ratio = 2.9492; 95% confidence interval = 1.0936–7.9537, *P *= .0326). However, no association was observed between NO_2_ and NOx exposure with COVID-19 (very severe respiratory confirmed). MR analysis demonstrates a potential causality between NO_2_ and NOx exposure and the risk of COVID-19. Preventing and controlling air pollution may help slow down and prevent the adverse progression of COVID-19.

## 1. Introduction

Coronavirus disease 2019 (COVID-19) is a global pandemic caused by the severe acute respiratory syndrome coronavirus 2 (SARS-CoV-2), known for its potential to induce severe respiratory symptoms and pulmonary lesions.^[1]^ To date, global confirmed cases have surpassed 760 million, with over 6.95 million recorded deaths.^[2]^

Air pollution denotes the contamination of the environment by chemical or biological agents, impacting the quality of the air inhaled by humans and other organisms. The World Health Organization identifies carbon monoxide (CO), particles with aerodynamic diameters of <10 μm (PM10) and <2.5 μm (PM2.5), ozone (O_3_), nitrogen dioxide (NO_2_) and sulfur dioxide (SO_2_) as the primary air pollutants affecting human health.^[3]^ Studies have demonstrated that air pollution induces airway inflammation and hyperresponsiveness, thereby increasing the incidence and severity of respiratory diseases.^[4–6]^

Nitrogen oxide (NOx) stands as a significant environmental pollutant, serving as a precursor to other air pollutants. Following a series of intricate reactions, NOx can undergo transformations to generate secondary organic and inorganic aerosols, thereby contributing to the formation of ground level O_3_ and fine particulate matter (PM2.5). The majority of environmental NOx originates from anthropogenic sources, such as fuel combustion, energy production, and emissions from road traffic vehicles.^[7–9]^ NOx appears primarily in 2 forms: NO_2_ and nitric oxide (NO). NO is typically discharged as a major pollutant and undergoes photochemical reactions with atmospheric free radicals to generate NO_2_. When no information is provided, it is reasonable to assume that the NO_2_ concentration is essentially equal to the NOx concentration, as any NO in the atmosphere rapidly transforms into NO_2_.^[10,11]^

Multiple epidemiological studies have suggested that air pollution may affect the spread, occurrence, and death of COVID-19, which may be linked to the dysregulated immune response associated with air pollutants and the enhancement of virus-induced tissue inflammation and damage.^[12–14]^ A recent Mendelian randomization (MR) study identified PM2.5 absorbance as a risk factor for COVID-19 infection, hospitalization, and severe respiratory symptoms.^[15]^ NOx is an important source of air pollutants other than particulate matter. Although limited cross-sectional studies have indicated a link between NO_2_ and the spread and fatality of COVID-19, consensus has yet to be established.^[16]^ Further investigation is warranted to validate the causal relationship between NOx and COVID-19 infection and hospitalization.

MR is a method that employs genotype as an instrumental variable to deduce the relationship between the phenotype and the disease. By doing so, it can mitigate the issue of reverse causality inference and provide insights into the long-term effects of exposure on outcomes. Hence, the aim of the present research was to utilize MR to examine the link between NOx and COVID-19 (infection, hospitalization, and severe respiratory symptoms) and offer insights into the development of COVID-19 prevention and control strategies.

## 2. Materials and methods

### 2.1. Study design

Causality and causality inference are fundamental considerations in epidemiological research. Observational epidemiology serves as a widely utilized method for causality inference, yet findings are frequently controversial owing to numerous challenges, including potential confounding factors and reverse causal associations. Randomized controlled trial is the most reliable method for causality inference in epidemiological studies. Nevertheless, their demanding requirements, rigorous control measures, and challenges in design and implementation, coupled with ethical considerations, often render direct utilization for studying disease causation challenging.

In an MR study, genetic variants known as single nucleotide polymorphisms (SNPs) are selected based on their strong correlation with exposure factors, serving as instrumental variables to represent the exposure factors under investigation. Through the analysis of the relationship between genetic variants and exposure factors, as well as genetic variants and outcomes, causality between exposure factors and outcomes can be inferred. The random allocation of alleles in this method bears similarity to that of RCTs.^[17,18]^ Furthermore, it enhances the directionality of causality and reduces reverse causality since the procedure cannot alter the genetic variants of exposure factors.

**Figure 1.**
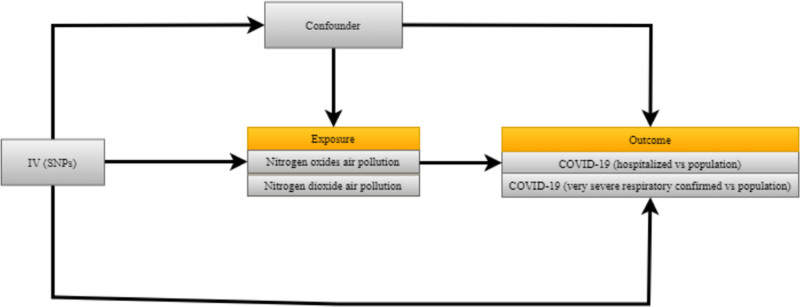
Study flowchart. Utilizing genetic variants as instrumental variables, MR analysis included three basic assumptions. The study needed to satisfy the following criteria: the genetic variants should be strongly linked to the exposure; independent of the confounding factors of the association between exposure and outcome; and only affect the outcome via the exposure. MR = Mendelian randomization.

Utilizing genetic variants as instrumental variables, MR analysis included 3 basic assumptions. The study needed to satisfy the following criteria (Fig. 1) the genetic variants should be strongly linked to the exposure; independent of the confounding factors of the association between exposure and outcome; and Only affect the outcome via the exposure.

### 2.2. Screening of genetic instrument

SNPs serving as instrumental variables linked to NOx air pollution were obtained from the integrative epidemiology unit (IEU) genome-wide association study (GWAS) dataset. These datasets are accessible through the IEU Open GWAS (GWAS ID: ukb-b-12417, and ukb-b-9942)^[17]^ (shown in Table 1). To identify the most robust instrumental variables, screening criteria (*P* < 5 × 10^−8^, clump: *r*^2^ = 0.001, kb = 10,000)^[18]^ were applied to the dataset to mitigate linkage disequilibrium, thereby leading to the exclusion of potential horizontal pleiotropy and nonsignificant SNPs.

**Table 1:** Data sources.

	Trait	Year	Consortium	Sample size	Number of SNPs	Population
Exposure						
ukb-b-12417	Nitrogen oxides air pollution	2018	MRC-IEU	4,56,380	98,51,867	European
ukb-b-9942	Nitrogen dioxide air pollution	2018	MRC-IEU	4,56,380	98,51,867	European
Outcome						
ebi-a-GCST011077	COVID-19 (very severe respiratory confirmed vs population)	2020	OPENgwas	10,59,456	74,96,658	European
ebi-a-GCST011084	COVID-19 (hospitalized vs population)	2020	OPENgwas	1206,629	75,34,178	European

COVID-19 = coronavirus disease 2019, IEU = integrative epidemiology unit, OPENgwas = open genome-wide association study, SNP = single nucleotide polymorphism.

### 2.3. COVID-19 data source

Summary data on COVID-19 case associations were obtained from the European Bioinformatics Institute complete GWAS summary database, accessible through the IEU Open GWAS.^[19]^ Two datasets were selected to investigate different COVID-19 statuses (shown in Table 1). Individuals who withdrew their consent from any one data source were excluded from the analysis.

### 2.4. Statistical analysis

Inverse-variance weighting (IVW) and MR using a robust adjusted profile score (RAPS) were employed for MR analysis.^[20,21]^ IVW, as the gold standard in MR, was the primary result in MR analysis. Fixed effects and random effects in IVW were used for analysis. In instances where the selected instrumental variables exhibited heterogeneity, the utilization of random effects IVW was deemed more robust. Conversely, in the absence of heterogeneity, fixed effects were considered more reliable. To mitigate weak instrument bias, the *F* statistic was utilized to gauge the intensity of instrumental variables. If the *F* exceeded 10, it was deemed that the results were not affected by weak instruments. If the *F* statistic of the instrumental variable approached 10, MR-RAPS was incorporated to supplement the analysis results. MR-RAPS, a method tailored to address weak instrumental variables, was employed to assist the results generated by IVW fixed effects.

In this study, NOx air pollution and NO_2_ air pollution were utilized as exposures, while COVID-19 (very severe respiratory confirmed vs population) and COVID-19 (hospitalized vs population) were considered as outcomes. Furthermore, a 2-sample MR was used to explore the causality. The overall odds ratio (OR) represented the effect of NOx on COVID-19, respectively. *P* < .05 were deemed as statistically significant values. Sensitivity analysis comprised 3 parts and various methods. Firstly, Cochran’s *Q* test value assessed heterogeneity. *P* < .05 depicted the presence of heterogeneity, although the multiplicative random effect model of the IVW method remained reliable in such cases.^[22]^ Secondly, horizontal pleiotropy was checked using MR-Egger regression^[23]^ to mitigate the second and third assumptions, with the MR-Egger intercept utilized for calculation. If the *P* value of the MR-Egger intercept was <.05, the effect of the SNP linked to the exposure factor on the results was considered unreliable. Thirdly, a leave-one-out analysis systematically excluded each SNP individually to assess if a single SNP notably altered the outcomes.^[24]^ Utilizing the IVW method, the “all” value was calculated, and the result was considered reliable if “all” >0. All analyses were executed utilizing R (v4.1.2) with the “TwoSampleMR”^[25]^ and “MR-PRESSO” packages.

## 3. Results

### 3.1. SNP screening

This study used NOx air pollution and NO_2_ air pollution as exposures, and COVID-19 (very severe respiratory confirmed vs population) and COVID-19 (hospitalized vs population) as outcomes; 2-sample MR was used to explore the causality (outcome and exposures can be searched at https://gwas.mrcieu.ac.uk/). In the UK Biobank study, *P* < 5E−08 was first used to screen exposed SNPs, and the number of SNPs that met the criteria was identified (second column of Table 1). Subsequently, linkage disequilibrium was addressed (third column of Table 1), and finally, outcome-associated SNPs were matched and screened to obtain the final set of SNPs (the fourth column of Table 2).

**Table 2:** Variable screening process.

	SNPs (*P* < 5 × 10^−8^)	Removal of existing linkage disequilibrium (LD)	Correct strand for non-palindromic SNPs
COVID-19 (very severe respiratory confirmed vs population)			
Nitrogen oxides air pollution	187	8	7
Nitrogen dioxide air pollution	241	8	5
COVID-19 (hospitalized vs population)			
Nitrogen oxides air pollution	187	8	8
Nitrogen dioxide air pollution	241	8	8

COVID-19 = coronavirus disease 2019, LD = linkage disequilibrium, SNP = single nucleotide polymorphism.

### 3.2. Pleiotropy and heterogeneity test

MR-Egger intercept test was utilized to check horizontal pleiotropy, with all *P* values observed above .05 (columns 4–5 of Table 3), indicating no evidence of horizontal pleiotropy. Similarly, when conducting the Heterogeneity Test neither of the 2 methods detected any heterogeneity, and all *P* values were >.05 (columns 6–9 of Table 3). Therefore, the fixed-effect IVW was utilized as the primary outcome in the MR analysis. Since COVID-19 (very severe respiratory confirmed vs population) was the outcome, the *F* value of NOx air pollution was 9.921 which was slightly <10. Consequently, MR-RAPs were used in the MR analysis to complement the results generated by the IVW fixed-effect.

**Table 3:** Hypothesis testing results.

	Strength		MR-Egger intercept test		Heterogeneity test			
	*F* value	*R*^2^ (%)	Intercept	*P*	*Q_* Egger	*P*	*Q*_IVW	*P*
COVID-19 (very severe respiratory confirmed vs population)								
Nitrogen oxides air pollution	9.921	0.015	−0.0307	.6622	1.3937	.925	1.6089	.9519
Nitrogen dioxide air pollution	10.752	0.012	0.288	.3746	1.5007	.6821	2.5833	.6298
COVID-19 (hospitalized vs population)								
Nitrogen oxides air pollution	11.343	0.020	−0.0379	.3602	3.096	.7967	4.077	.7709
Nitrogen dioxide air pollution	10.306	0.018	−0.0272	.5152	1.0511	.9836	1.529	.9813

COVID-19 = coronavirus disease 2019, IVW = inverse-variance weighting, MR = Mendelian randomization.

### 3.3. MR analysis of NOx air pollution on the status of COVID-19

After completing the test, MR analysis was initiated with the screened SNPs. Among them, with COVID-19 (hospitalized vs population) as the outcome, the IVW fixed-effect of the OR (95% confidence interval [CI]) of NOx air pollution was found to be: 3.7609 (1.2726–11.1144), with the interval not including 1 and *P* = .0166. This suggests that NOx air pollution was positively correlated with the risk of COVID-19 (hospitalized vs population), and both IVW random effect model and MR-RAPs showed similar results. In addition, the IVW fixed-effect of the OR (95% CI) of NO_2_ air pollution was observed at 2.9492 (1.0936–7.9537), with its interval not including 1 and *P* = .0326. It indicated that NOx air pollution and NO_2_ air pollution were positively correlated with the risk of COVID-19 (hospitalized vs population), and both IVW random effect model and MR-RAPs showed similar results (Table 4).

**Table 4:** The MR results.

	Method	OR (95% CI)	*P*-value
COVID-19 (very severe respiratory confirmed vs population)			
Nitrogen oxides air pollution	IVW (random effect)	3.3432 (1.1902–9.3913)	.0220
	IVW (fixed-effect)	3.3432 (0.4549–24.5684)	.2356
	MR-RAPS	3.3709 (0.4152–27.3682)	.2554
Nitrogen dioxide air pollution	IVW (random effect)	1.0609 (0.1754–6.4156)	.9486
	IVW (fixed-effect)	1.0609 (0.1130–9.9589)	.9587
	MR-RAPS	1.0617 (0.1031–10.9343)	.9599
COVID-19 (hospitalized vs population)			
Nitrogen oxides air pollution	IVW (random effect)	**3.7609 (1.6449–8.5987**)	**.0017**
	IVW (fixed-effect)	**3.7609 (1.2726–11.1144**)	**.0166**
	MR-RAPS	**3.8389 (1.2327–11.9551**)	**.0203**
Nitrogen dioxide air pollution	IVW (random effect)	**2.9492 (1.8550–4.6890**)	**<.0001**
	IVW (fixed-effect)	**2.9492 (1.0936–7.9537**)	**.0326**
	MR-RAPS	**2.9689 (1.0461–8.4260**)	**.0409**

Statistical significance was defined as *P* < .05.

CI = confidence interval, COVID-19 = coronavirus disease 2019, IVW = inverse-variance weighting, MR = Mendelian randomization, OR = odds ratio, RAPS = robust adjusted profile score. Statistical significance was defined as *P* < 0.05.

However, when considering COVID-19 (very severe respiratory confirmed vs population) as the outcome, the analysis found that the causality between NOx air pollution, NO_2_ air pollution and COVID-19 (very severe respiratory confirmed vs population) was negative. The OR (95% CI) of NOx air pollution was 3.3432 (0.4549–24.5684), and the interval included 1, and the *P* value was >.05. The OR (95% CI) of NO_2_ air pollution was 1.0609 (0.1754–6.4156), and the interval included 1, and the *P* value was >.05. The data acquired suggested that there was no significant causality between NOx air pollution, NO_2_ air pollution and COVID-19 (very severe respiratory confirmed vs population). Based on the results of NOx air pollution, NO_2_ air pollution and COVID-19 (hospitalized vs population), it demonstrated that NOx air pollution and NO_2_ air pollution were associated with COVID-19, but no association with extremely severe COVID-19 cases was noted.

**Figure 2.**
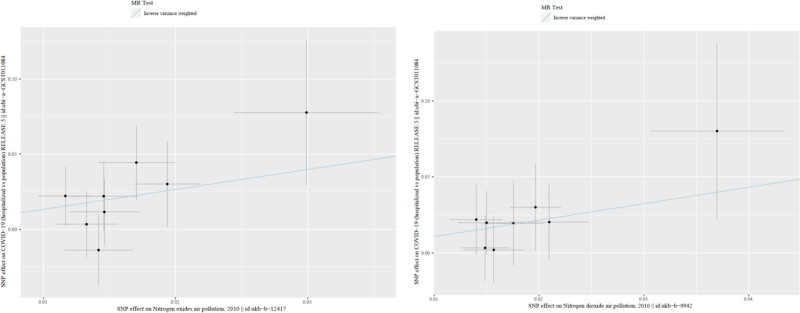
Scatter plot. Draw scatter plots of the 2 exposures of nitrogen oxides air pollution and nitrogen dioxide air pollution and COVID-19 (hospitalized vs population), and the blue line can be seen to tilt upward, indicating a positive correlation effect. COVID-19 = coronavirus disease 2019.

**Figure 3.**
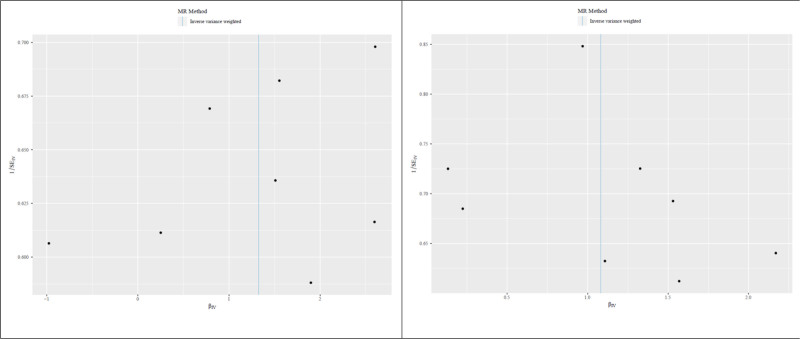
Funnel plot. The funnel plot shows the partial middle position of the blue line of all SNP scatter points, indicating the small selective heterogeneity of SNPs. SNP = single nucleotide polymorphism.

**Figure 4.**
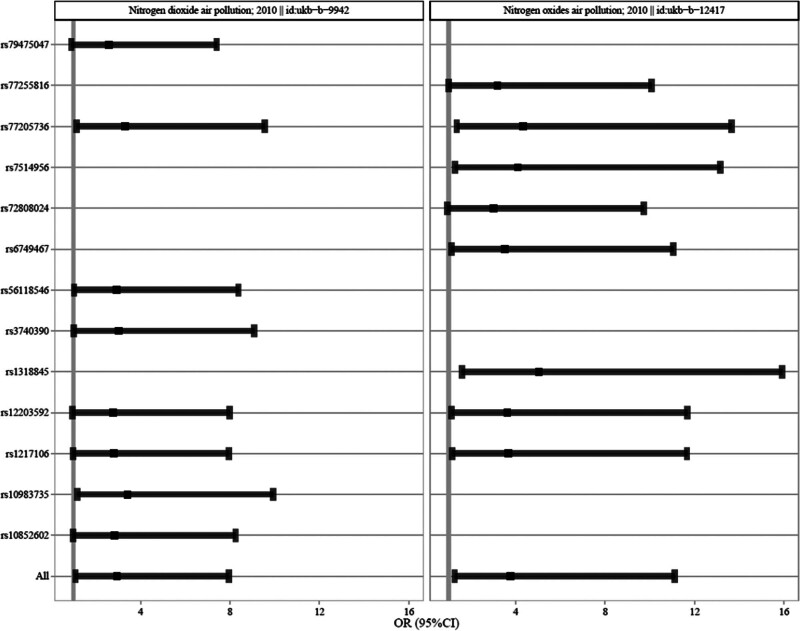
A leave-one method sensitivity analysis. Then each scatter center was >1 after removing each SNP individually, further demonstrating the robustness of the positive correlation. SNP = single nucleotide polymorphism.

To further elucidate the robustness of the positive results, the visual scatter plot was utilized. In Figure 2, the fitting line (blue line) of IVW pointed upward, namely the slope was positive. It further showed that NOx air pollution and NO_2_ air pollution were positively correlated with the risk of COVID-19 (hospitalized vs population). In addition, the funnel plot was utilized (Fig. 3) to show that there was no heterogeneity in the selected SNP, specifically the more uniformly the scatter points were distributed on the left and right sides of the blue line, the smaller the heterogeneity. Moreover, leave-one-out analysis was utilized as a sensitivity measure to ensure the reliability of the positive results and mitigate the risk of false positives stemming from the disproportionate influence of individual SNPs (Fig. 4). As shown in Figure 4, as each SNP was successively removed, the IVW result point estimates (black blocks in the line segments) of the remaining SNPs consistently fell on the right of OR = 1 (the vertical gray line was the dividing line of OR = 1), namely OR value > 1. This pattern reaffirmed the robustness of our findings, providing additional support for the potential causality between NOx air pollution, NO_2_ air pollution, and the risk of COVID-19 (hospitalized vs population).

## 4. Discussion

In this investigation, pertinent whole-genome data was acquired from GWAS to be utilized as genetic tools in exploring the potential causality between NOx exposure and COVID-19 outcomes, including infection, hospitalization, and severe respiratory symptoms. Our analysis revealed a potential association between NO_2_ and NOx exposures and the heightened risk of hospitalization due to COVID-19, however, the wider confidence interval (1.27–11.11) indicates that there is considerable uncertainty in the estimated results. This emphasizes the need for caution when interpreting the results, as the wider confidence interval limits the precision of our findings. The results section further discusses possible sources of uncertainty, such as sample variability and measurement error. Further support for this positive association was found through additional analyses, encompassing scatter plot, funnel plot, and leave-one-out sensitivity analyses. These analyses examined the influence of NOx and NO_2_ air pollution on the risk of COVID-19 (hospitalized vs population). It was observed that NOx air pollution or NO_2_ air pollution did not exhibit a significant association with critical respiratory symptoms of COVID-19, although our study did not find a significant association between NOx or NO_2_ exposure and severe respiratory COVID-19, this may indicate that air pollution influences hospitalization decisions rather than directly affecting disease severity. The lack of a significant association may reflect the influence of factors such as access to medical resources and clinical management decisions, which are further discussed in the discussion section. Therefore, while air pollution is associated with COVID-19 hospitalization, it may not directly determine the clinical outcome of the disease.

Outdoor air pollution comprises various pollutants, with NOx being one of the most significant contributors, and NO_2_ specifically exerts detrimental effects on the respiratory system.^[26]^ It has been observed that long-term exposure to NO_2_ is linked to a range of serious diseases, including diabetes, cardiovascular diseases, and hypertension, and may lead to death in certain cases.^[27,28]^ Exposure to NO_2_ stimulates human bronchial epithelial cells, promoting the release of inflammatory factors and inducing oxidative stress. In addition, exposure to NO_2_ enhances the adhesion of neutrophils to exposed airway epithelial cells, resulting in the death of the exposed cells.^[29]^ Similarly, NO_2_, as an outdoor anthropogenic source (particularly originating from the transport of pollutant particles associated with fossil energy combustion) has the potential to exacerbate the progression of COVID-19 and lead to adverse outcomes.^[30,31]^ Experimental exposure to NO_2_ has been shown to increase susceptibility to influenza A virus and respiratory syncytial virus infections.^[32,33]^ Recent evidence suggests that NO_2_ can promote the susceptibility of the respiratory system to SARSCoV-2 infection and indicates a significant correlation between NO_2_ concentrations and the spread of COVID-19.^[16,34,35]^ In addition, the detrimental effects of NOx on other bodily systems may exacerbate the severity of COVID-19, potentially leading to COVID-19-linked fatalities.

Multiple epidemiological investigations worldwide have highlighted the associations between NOx and COVID-19 morbidity and mortality. For instance, studies conducted in India by Chakraborty et al^[35]^ showed that NO_2_ concentrations had a statistically positive correlation with COVID-19 deaths and case fatality rates across 18 Indian states. The data acquired here suggest that people who were frequently exposed to vehicle exhaust might face higher risks in the COVID-19 pandemic. In a large observational study conducted from January 23, 2020 to February 29, 2020, in China, Zhu et al^[36]^ collected daily confirmed cases, air pollution concentrations, and meteorological variables across 120 cities. Utilizing a generalized additive model, the researchers found that the concentration of NO_2_ was significantly positively correlated with new confirmed cases of COVID-19. Specifically, they observed that a 10-μg/m^3^ increase in NO_2_ was linked to a 6.94% rise in daily COVID-19 cases. Yao et al^[37]^ reported significant association between variables through cross-sectional and longitudinal analyses, indicating that environmental NO_2_ might have significantly contributed to the expansion of the COVID-19 pandemic in Hubei Province. Catalonia, one of the hardest-hit regions in Spain during the COVID-19 pandemic, was the focus of a mixed longitudinal ecological study conducted by Saez et al^[38]^ The study aimed to determine whether the risk of COVID-19 morbidity and mortality was influenced by prolonged exposure to air pollutants such as NO_2_. The investigation spanned from February 25, 2020 to May 16, 2020. The acquired data revealed for NO_2_, for every 1 μm/m^3^ above the mean, the risk of a positive result increased by 2.7%. Marqu`et al^[39]^ also identified an association between COVID-19 and NO_2_ in Catalonia in 2021. Additionally, studies by Travaglio et al^[40]^ showed that NOx levels in the UK were significantly associated with COVID-19 deaths. Filippini et al^[41]^ investigated the prevalence of infection in the heavily impacted regions of 28 northern Italian provinces. Their study involved gathering tropospheric NO_2_ levels using satellite data provided by the European Space Agency. A significant positive correlation between NO_2_ levels and SARS-CoV-2 prevalence was found in areas with higher NO_2_ levels. In addition, Ogen^[42]^ investigated the association between NO_2_ concentrations and COVID-19 deaths measured across 66 administrative regions in 4 European countries (Italy, France, Spain, and Germany). By controlling for atmospheric conditions, it was found that 78% of deaths occurred in areas with the highest NO_2_ concentration levels. Consequently, areas with higher NO_2_ concentrations exhibited significant association with COVID-19-related fatality rates.

This study utilized the IVW method revealing that NO_2_ concentration might function as a risk factor for COVID-19 hospitalization. Moreover, various statistical models: fixed-effect IVW, IVW random effect model and MR-RAPs, suggested that NO_2_ pollution might heighten the risk of COVID-19 infection. In addition, visual scatter plot, funnel plot, and leave-one-out method were employed to further confirm the robustness of the positive results. This study represents the first attempt at utilizing MR analysis to investigate the causality between NOx and COVID-19, providing a reference for COVID-19 prevention and control. Furthermore, compared to observational studies, the MR method minimizes the influence of confounding factors and reverse causality on the association analyses.

Although the MR method is suitable for assessing the causal effects of genetically determined exposures, environmental pollution exposures are influenced by external factors such as lifestyle, place of residence, and socioeconomic status. The genetic tools we selected as proxies for long-term pollution exposure may not fully capture actual environmental exposures, which vary depending on individual behavior and external conditions. We recognize this limitation and further emphasize the importance of considering these factors in interpreting the study results in the discussion section. Future studies incorporating more precise exposure data could help clarify this relationship.

We recognize that factors such as socioeconomic status, occupation, urbanization, and comorbidities, which have a significant impact on air pollution exposure and COVID-19 risk, were not adequately considered in this study. To mitigate this limitation, we used genetic tools, which are relatively less susceptible to these confounding factors; however, we are aware that these external factors may still have some residual influence. Future studies should more rigorously control for these factors to better exclude confounding biases and accurately assess the causal effects of air pollution on COVID-19 outcomes.

Despite its strengths, this study is limited in certain respects. Firstly, the GWAS data utilized in the analyses are limited to European populations, restricting our ability to evaluate the impact of sex and age on the observed associations in summary data. Consequently, genetic data from diverse regions, races, and settings are lacking, limiting the generalization of these findings to other populations or contexts. Secondly, we assessed heterogeneity and horizontal pleiotropy and encountered constraints due to the limited number of SNPs included in our analysis. These limitations undermine the reliability of the association between PM2.5 and COVID-19 infection. The heterogeneity observed may stem from variations in the detection methods utilized to obtain the relevant data. While the IVW method may still offer reliable insights in certain scenarios,^[43]^ it is crucial to identify the instrumental variables or procure new GWAS data to mitigate the influence of horizontal pleiotropy. In addition, the limited availability of genetic tools may lead to higher standard errors in statistical models thereby impacting their accuracy, underscoring the importance of regularly updating relevant GWAS data. Third, the assessment of year-round exposure to outdoor air pollution levels from UKB sources overlooked the potential impacts of indoor environmental pollutants. Additionally, the emergence of more virulent SARS-CoV-2 variants in different geographical areas post-2021^[44,45]^ was not accounted for as our data were collected in 2020. Consequently, data on the adverse effects of new virus strains on individuals or genes were not included, suggesting that incorporating the latest data can further improve the association. Furthermore, environmental pollution parameters such as NO_2_ require further analysis through linear or nonlinear MR studies to obtain more reliable findings.

## 5. Conclusions

MR analysis demonstrates a potential causality between NO_2_ and NOx exposure and the risk of COVID-19. Preventing and controlling air pollution may help slow down and prevent the adverse progression of COVID-19.

## Author contributions

**Conceptualization:** Jing Cao, Haibo Xu, Zixiao Chen, Jiaona Wei.

**Data curation:** Jing Cao, Haibo Xu, Kun He, Jiaona Wei.

**Formal analysis:** Jing Cao, Zixiao Chen, Kun He, Jiaona Wei.

**Funding acquisition:** Haibo Xu, Zixiao Chen.

**Investigation:** Haibo Xu, Kun He, Jiaona Wei.

**Methodology:** Jing Cao, Haibo Xu.

**Project administration:** Jing Cao, Zixiao Chen, Kun He.

**Resources:** Jing Cao, Haibo Xu, Kun He, Jiaona Wei.

**Software:** Jing Cao, Haibo Xu, Zixiao Chen, Kun He, Jiaona Wei.

**Supervision:** Jing Cao, Haibo Xu, Zixiao Chen, Kun He.

**Validation:** Jing Cao, Zixiao Chen, Kun He.

**Visualization:** Jing Cao, Haibo Xu, Jiaona Wei.

**Writing** – **original draft:** Jing Cao, Haibo Xu.

**Writing** – **review & editing:** Jiaona Wei.
